# One-Pot Synthesis of N-Rich Porous Carbon for Efficient CO_2_ Adsorption Performance

**DOI:** 10.3390/molecules27206816

**Published:** 2022-10-12

**Authors:** Qiyun Yu, Jiali Bai, Jiamei Huang, Muslum Demir, Bilge Nazli Altay, Xin Hu, Linlin Wang

**Affiliations:** 1 Key Laboratory of the Ministry of Education for Advanced Catalysis Materials, Zhejiang Normal University, Jinhua 321004, China; 2 Department of Chemical Engineering, Osmaniye Korkut Ata University, Osmaniye 80000, Turkey; 3 College of Engineering Technology, Print and Graphic Media Science, Rochester Institute of Technology, Rochester, NY 14623, USA; 4 Institute of Pure and Applied Sciences, Marmara University, Istanbul 34722, Turkey; 5 Key Laboratory of Urban Rail Transit Intelligent Operation and Maintenance Technology and Equipment of Zhejiang Province, College of Engineering, Zhejiang Normal University, Jinhua 321004, China

**Keywords:** porous carbon, CO_2_ adsorption, one-pot KOH activation, N-doped

## Abstract

N-enriched porous carbons have played an important part in CO_2_ adsorption application thanks to their abundant porosity, high stability and tailorable surface properties while still suffering from a non-efficient and high-cost synthesis method. Herein, a series of N-doped porous carbons were prepared by a facile one-pot KOH activating strategy from commercial urea formaldehyde resin (UF). The textural properties and nitrogen content of the N-doped carbons were carefully controlled by the activating temperature and KOH/UF mass ratios. As-prepared N-doped carbons show 3D block-shaped morphology, the BET surface area of up to 980 m^2^/g together with a pore volume of 0.52 cm^3^/g and N content of 23.51 wt%. The optimal adsorbent (UFK-600-0.2) presents a high CO_2_ uptake capacity of 4.03 mmol/g at 0 °C and 1 bar. Moreover, as-prepared N-doped carbon adsorbents show moderate isosteric heat of adsorption (43–53 kJ/mol), acceptable ideal adsorption solution theory (IAST) selectivity of 35 and outstanding recycling performance. It has been pointed out that while the CO_2_ uptake was mostly dependent on the textural feature, the N content of carbon also plays a critical role to define the CO_2_ adsorption performance. The present study delivers favorable N-doped carbon for CO_2_ uptake and provides a promising strategy for the design and synthesis of the carbon adsorbents.

## 1. Introduction

Burning fossil fuels for electricity, heat and transportation is the primary reason for greenhouse gas emissions from human and industrial activities. These gases hold heat in the atmosphere and cause global warming, especially CO_2_ contributes more than 60–70% [1]. It is one of the most disastrous environmental problems and great interest to strip as much as CO_2_ from industrial waste gases or the atmosphere for the serenity of global warming. Carbon capture and sequestration/storage technologies are considered to play a key role in reducing the emission [2,3,4]. Current post-combustion CO_2_ capture technologies that have high repeatability and selectivity include the chemical absorption techniques like amine scrubbing [5], ionic liquid absorption [6] and the adsorption techniques that use physical adsorbents [7,8] or amine-, lithium- or calcium-based chemical adsorbents [9,10].

Various physical adsorbents such as carbonaceous material [11,12,13,14,15,16,17,18,19,20,21], zeolite [22], ordered mesoporous silica [23], metal-organic frameworks (MOFs) [24,25,26], porous polymers [27,28,29] and membrane-based systems [30] have been investigated to capture CO_2_. Among these, porous carbons have been receiving significant attention for their wide-scale availability, ease of regeneration, low cost, high chemical/thermal stability, large surface area and capability of being tuned for applications not only for adsorbents but also for supercapacitors, battery electrodes and catalyst supports [31,32,33,34,35,36]. Moreover, the process of using porous materials for CO_2_ adsorption has been reported to have less energy requirements thanks to the lower adsorption energy needed relative to absorption processes. Typically, low-temperature ranges (<473 K) were reported for adsorbents such as MOFs, zeolites, silica and carbons; while intermediate range (473–673 K) for metal oxides and high-temperature range (>673 K) for lithium zirconate [37]. The interaction between the CO_2_ and the wide pore surface of porous carbon is found to be the parameter of the adsorption feature, called isotherm, which is improved by controlling the synthetic conditions and the kind of precursor. Using heteroatoms like nitrogen (N) and oxygen to dope porous carbon is also found to improve a surface property, selectivity and adsorption capability due to the enhanced acid-base, quadrupolar and/or hydrogen bonding interactions [15,18,33,35,38,39,40,41,42]. Besides conventional solid adsorbents, 3D printed polymer composites have also been studied that enable high CO_2_ capture using a direct ink writing method to intricate specified properties like porosity [43]. Previous studies have pointed out the importance of narrow micropores (<1 nm) that allow higher CO_2_ adsorption and CO_2_/N_2_ selectivity [44,45]. The minimum CO_2_ capture capacity of 2 mmol/g and >100 CO_2_/N_2_ selectivity are reported to be desirable for an adsorbent to be satisfactory [37].

Current research has been focusing on improving selectivity and the adsorption capacity of CO_2_ by using different precursors or by making different structures [46,47,48]. A recent paper concluded that porous carbon is cost-efficient material, but additional research is needed to investigate its full potential by modifying experiments to optimize such textural properties and CO_2_-philic heteroatom doping (like N, S) [37]. However, the synthetic routes of heteroatom-doped porous carbons usually involve multiple steps such as pre-carbonization, post heteroatom-doping and chemical activation [15,16,49]. This multiple-step synthesis method is considered not only highly energy consuming but also low production yield. Therefore, in the present study, we are aimed to produce N-doped porous carbon with one step approach, which is the main novelty part of the present work.

In this work, a commercial UF resin is used as the precursor to synthesize nitrogen-doped porous carbonaceous CO_2_ adsorbent via a facile one-step KOH carbonizing method under various conditions. The effect of the KOH/UF mass ratios on elemental, textural and surface properties has been characterized and reported along with the CO_2_ capture performance, CO_2_/N_2_ selectivity, dynamic CO_2_ capture capacity.

## 2. Results and Discussion

### 2.1. Morphological, Phase Structural, and Surface Chemical Properties

**Figure 1:**
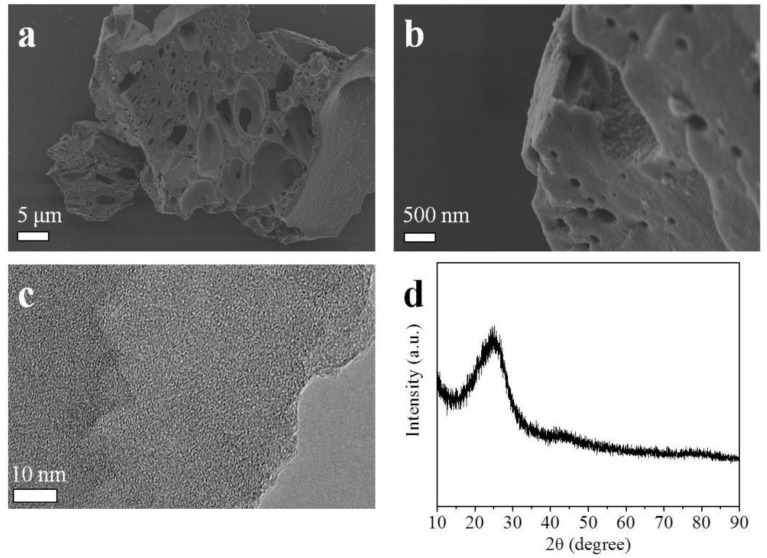
SEM images (**a**,**b**), TEM image (**c**) and XRD pattern (**d**) of UFK-600-0.2.

The scanning electron microscopy (SEM) and transmission electron microscopy (TEM) analyses were employed to explore the morphology of the representative UFK-600-0.2 sample. Figure 1a,b depict the 3D continuous block-shaped morphology that contains micropores with different pore sizes aligned with the interconnected channels. Figure 1c shows the TEM image of UFK-600-0.2 further revealing the porous structure with the random worm-like micropores on the carbon with pore sizes from a few dozen nanometers to a micrometer. A similar structural network was reported previously to be significant for CO_2_ gas uptake. The X-ray diffraction (XRD) pattern of the UFK-600-0.2 given in Figure 1d shows the typical diffraction peaks at around 2θ = 25 and 43° that are ascribed to the diffraction of the (002) and (100) crystal planes of the graphitic carbon [50].

**Figure 2:**
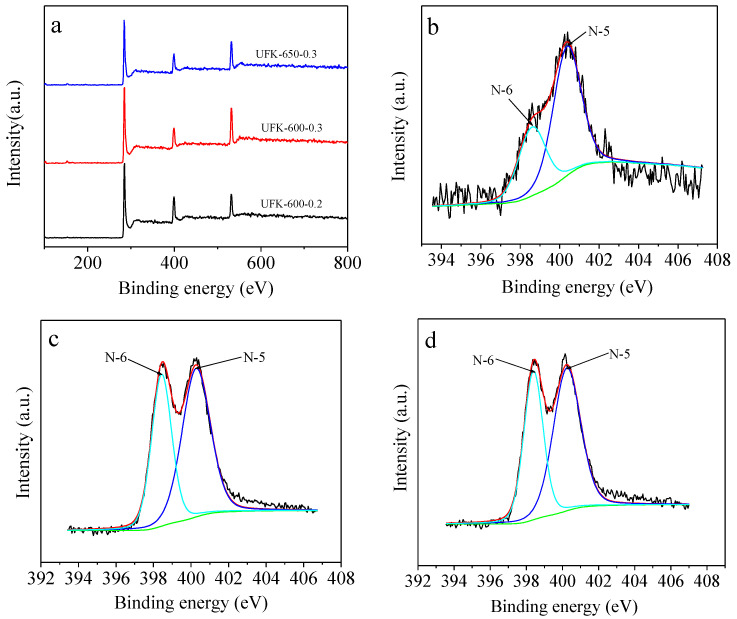
XPS survey (**a**) of selected adsorbents, XPS N1s of (**b**) UFK-600-0.2, (**c**) UFK-600-0.3, and (**d**) UFK-650-0.3.

Based on the elemental analysis results, these urea formaldehyde resin-derived N-doped porous carbons possess exceptional high N content ranging from 16.85 wt% to 23.51 wt%, as listed in Table 1. With the increasing of activation temperature and KOH/UF ratio, the N content of the as-synthesized carbons decreased, which is consistent with previous studies. To further study the nature of N present on the carbon surface, X-ray photoelectron spectroscopy (XPS) analysis was employed on the selected representative UFK-600-0.2, UFK-600-0.3 and UFK-650-0.3 adsorbents. From the survey plot in Figure 2a, all selected adsorbents are mostly composed of C, N, and O elements. In the case of N1s deconvolution spectra (Figure 2b–d), two main peaks representing pyridinic N (N-6) and pyrrolic N (N-5) were found for these samples. Those binding energies for pyridinic N and pyrrolic N groups were located at 398.4 and 400.2 eV, respectively [51]. Quantitative analysis found that the amount of N-5 is higher than that of N-6 for these samples (Table S1). Previous studies have suggested that pyrrolic nitrogen was possibly the highest beneficial anchor site for CO_2_ adsorption [52,53]. Thus, there UFK-T-m carbons are appropriate adsorbents for CO_2_ adsorption applications.

### 2.2. Porous Textual Properties

**Figure 3:**
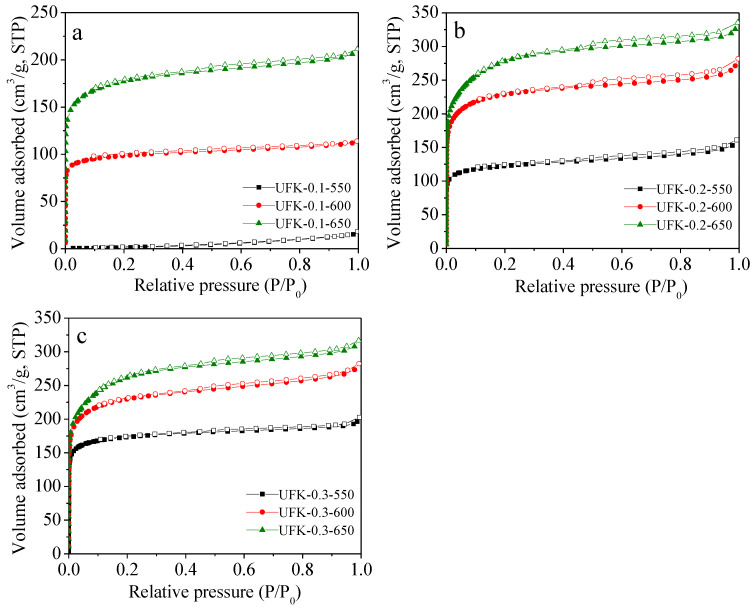
N_2_ sorption isotherms of the samples prepared at KOH/UF mass ratio of (**a**) 0.1, (**b**) 0.2 and (**c**) 0.3. Filled and empty symbols represent adsorption and desorption branches, respectively.

**Figure 4:**
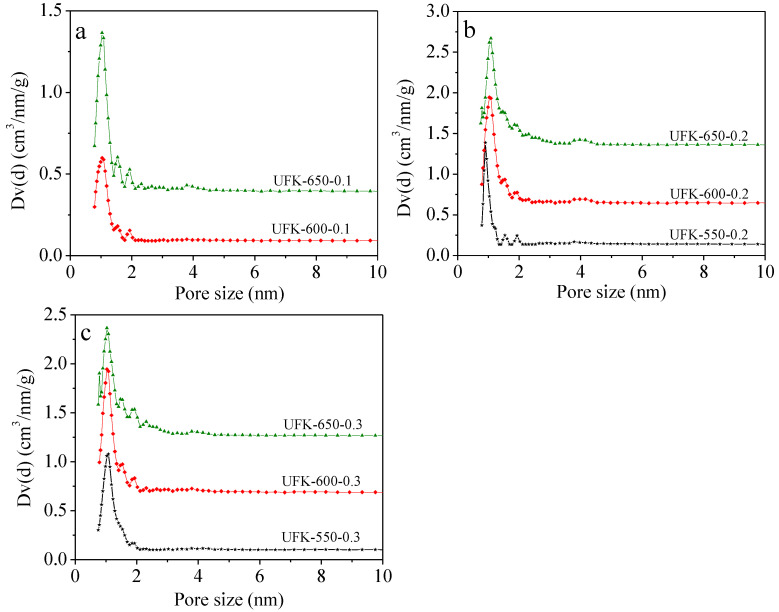
Pore size distribution of the samples prepared at KOH/UF mass ratio of (**a**) 0.1, (**b**) 0.2 and (**c**) 0.3. Due to the almost non-porous nature of UFK-550-0.1, its PSD is not shown here.

The detailed textural parameters derived from N_2_ sorption at 77 K including the Brunner−Emmet−Teller (BET) surface area (S_BET_), total pore volume (V_0_) and micropore volume (V_t_) were listed in Table 1. The N_2_ adsorption and desorption isotherms of as-prepared porous carbons under different conditions are shown in Figure 3. For all adsorbents, the high amount of N_2_ adsorption observed at the low relative pressure region (*P/P_0_* < 0.01) except for UFK-550-0.1 indicate the typical type-I curve according to the IUPAC classification, which refers to the numerous existence of micropores structure. It is worth noting that a wide knee curvature was observed for the UFK-650-0.1, UFK-650-0.2, UFK-600-0.2, UFK-600-0.3 and UFK-650-0.3 adsorbents signifying the existing of small mesopores or macropores. As seen from the pore size distribution (PSD) in Figure 4, multi-mode pore sizes were presented where most pores were less than 2.0 nm and a small percentage of pores in the 2–10 nm range also formed, further support existing of micro and meso porosities within the carbon matrix [54]. Almost all UFK-T-m adsorbents (expect UFK-550-0.1) depicted well-developed pore structure, where the surface areas and pore volumes were found in the range of 388–950 m^2^/g and 0.18–0.49 cm^3^/g, respectively. To explore the effect of the KOH amount on the pore formation, we investigated three KOH/UF mass ratios i.e., 0.1, 0.2 and 0.3 and the trend was found that as the KOH/UF mass ratio increased, the pore-development of carbon enhanced. To our curiosity, we also examine the effect of activating temperature on the development of textural properties of carbon. Generally speaking, as the activating temperature rise from the 550 to 650 °C, all textural properties including the BET surface area and total pore volume were significantly increased owing to the lower activating temperature was unfavorable for pore development since the thermal energy is not sufficient to etching of the carbon matrix at relatively low temperature. In short, the optimal sample was found as UFK-650-0.3 considering the textural feature of as-prepared carbons. However, it is critical to note that the highest textural performance is not a single parameter to determine optimal CO_2_ adsorption capacity as discussed in the next section [55,56].

### 2.3. CO_2_ Adsorption Analysis for the Porous Carbons

**Figure 5:**
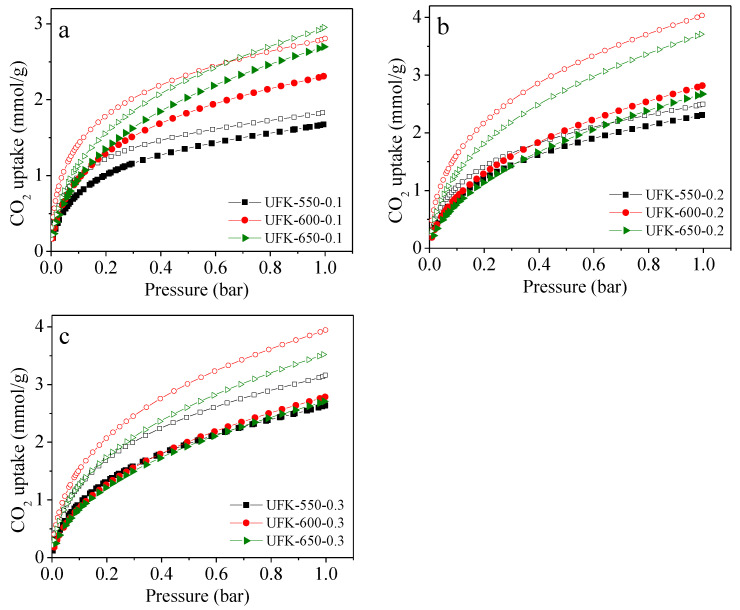
CO_2_ adsorption isotherms at 25 °C (filled) and 0 °C (empty) for urea formaldehyde resin-derived N-doped carbons prepared under KOH/UF mass ratio of (**a**) 0.1, (**b**) 0.2 and (**c**) 0.3.

**Figure 6:**
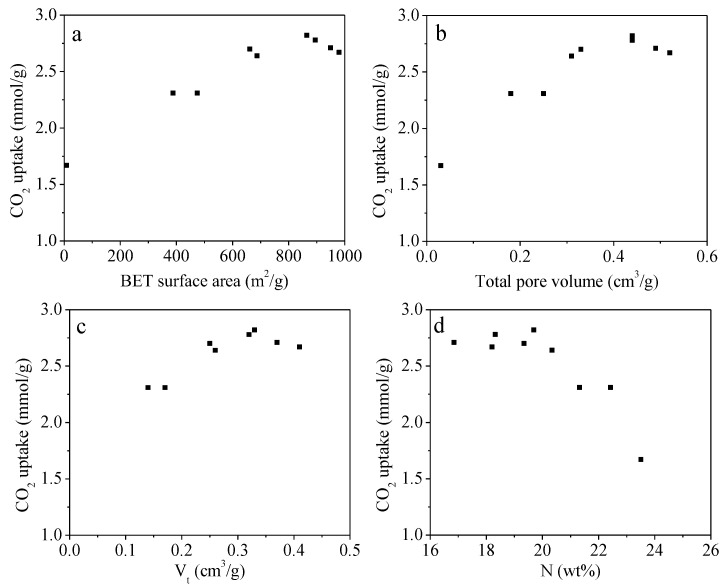
Plot of each porous properties characteristics (**a**) S_BET_, (**b**) V_0_, (**c**) V_t_ and (**d**) nitrogen content versus CO_2_ uptake at 25 °C and 1 bar.

As indicated above, having advanced textural properties, 3D block-shaped morphology and highly rich nitrogen functionality, as-prepared UFK-T-m materials are remarkable to investigate CO_2_ capture performance. The CO_2_ adsorption isotherms of UFK-T-m adsorbents at 0 and 25 °C were shown in Figure 5. It has been noticed that the CO_2_ adsorption capacity was not level off even at a pressure of 1 bar, signifying excess CO_2_ uptake capacity could be obtained at higher pressures, which indicates of physisorption mechanism. The CO_2_ capture capacities of the adsorbents were given in Table 1. At 0 °C, considering the KOH/UF mass ratios i.e., 0.1, 0.2 and 0.3, it is significant that the CO_2_ uptake capacity reached the highest number when the KOH/UF mass ratios of 2 in all activating temperatures. Moreover, we further explore the effect of the activating temperature on the CO_2_ uptake capacities and found that the carbon capture capacity was increased from 2.50 to 4.03 mmol/g as the activating temperature rise from 550 to 600 °C but the capacity dropped from the 4.03 to 3.71 mmol/g. The same trend has been found when the adsorption temperature was set to 25 °C. In short, based on the 9 trial analysis, the UFK-600-0.2 sample (activated at 600 °C with the KOH/UF mass ratios of 2) was found to be an optimal adsorbent taking considering into the CO_2_ capture performance. Please note that we fully analyzed S_BET_, V_0_, V_t_ and nitrogen content versus CO_2_ uptake performance and shown in Figure 6. To our curiosity, we speculate the scientific reasons behind the optimal sample. It is hard to understand that the optimal UFK-600-0.2 adsorbent has not either the highest BET surface area or nitrogen content. It means that the textural feature and nitrogen functionality content have both decided the CO_2_ capture activity of the N-doped carbons. What is more, the UFK-650-0.2 with the highest BET surface area along with the largest pore volume did not depict the largest adsorption capacity while the UFK-550-0.1 sample presented poor CO_2_ uptake capacity though it has the largest nitrogen content of 23.51 wt%, signifying that neither the total porosity or nitrogen content is a single factor in defining the CO_2_ uptake performance. Thus, the overall results show that the combination of textural features in particular micro/mesopores and suitable nitrogen functionality of optimal UFK-600-0.2 sample contribute a positive effect on CO_2_ uptake capacity. A similar phenomenon was previously reported in the literature [57,58,59]. It is worth pointing out that the optimal adsorbent UFK-600-0.2 in this study possesses fair CO_2_ capture performance compared to many typical adsorbents such as porous carbons [60,61], MOFs [24], COFs [62], porous aromatic frameworks (PAFs) [63] and porous polymers [27]. A comparison of the CO_2_ adsorption capacity among different adsorbents can be found in Table S2 (Supplementary materials).

**Figure 7:**
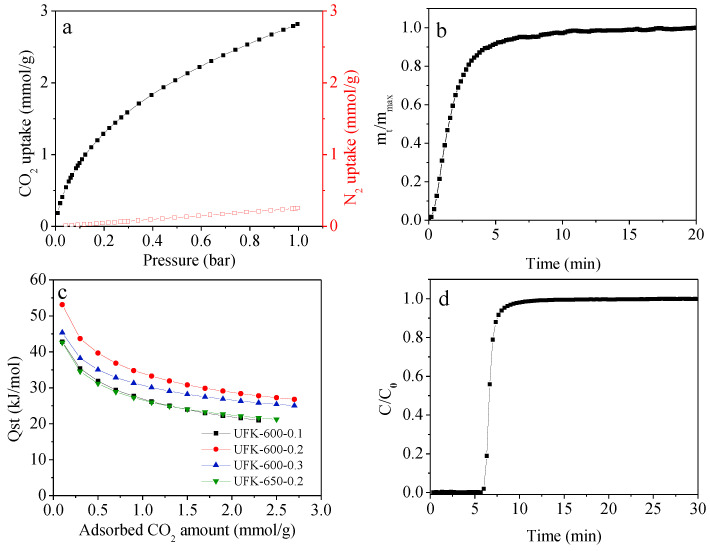
(**a**) CO_2_ and N_2_ adsorption isotherms of UFK-600-0.2 at 25 °C and 1 bar, (**b**) CO_2_ adsorption kinetics at 25 °C for UFK-600-0.2, (**c**) Qst on selected sorbents and (**d**) breakthrough curves of UFK-600-0.2. Adsorption conditions: gas pressure 1 bar, adsorption temperature 25 °C, gas flow rate 10 mL/min, inlet CO_2_ concentration 10 vol.%.

The CO_2_ and N_2_ adsorption isotherms of UFK-600-0.2 at 25 °C and 1 bar were shown in Figure 7a. Applying the ideal adsorption solution theory (IAST) [64], the selectivity of CO_2_ over N_2_ was calculated in a mixture of CO_2_ (0.10 bar) and N_2_ (0.90 bar). The IAST selectivity of CO_2_/N_2_ was found to be 35 for the optimal UFK-600-0.2 sample owing to high micropore volume and the presence of high nitrogen functionality on the carbon surface.

As a promising CO_2_ adsorbent, apart from high CO_2_ capture capacity and selectivity, the kinetics of adsorption must be rapid. To define the CO_2_ capture kinetic feature, the kinetic performance of the optimal UFK-600-0.2 was inspected at 25 °C. As shown in Figure 7b, 90% of the CO_2_ adsorption saturated was observed, signifying its quick CO_2_ uptake rate [65].

Isosteric heat of adsorption (Q_st_) is another critical parameter to determine the interaction strength between the CO_2_ molecules and solids adsorbents [66]. As shown in Figure 7c, the selected adsorbents depict the initial Q_st_ values in the range of 43–53 kJ/mol, which refer to relatively strong physisorption progress, most probably owing to a large amount of nitrogen functionality within the carbon framework. This is further supported by the declining Q_st_ values with the increasing CO_2_ loading, which signifies the chemistry surface of the N-doped adsorbents and the surface heterogeneity [67].

To assess the realistic separation performance of CO_2_ over N_2_, a breakthrough experiment was conducted for the optimal UFK-600-0.2, where the adsorption conditions were the gas mixture CO_2_/N_2_ (10:90) with a flow rate of 10 mL min-1 at 25 °C. Based on the breakthrough curves of UFK-600-0.2 in Figure 7d, the CO_2_ dynamic capture capacity was found to be 0.85 mmol/g signifying an outstanding perspective in CO_2_ adsorption from the flue gas.

**Figure 8:**
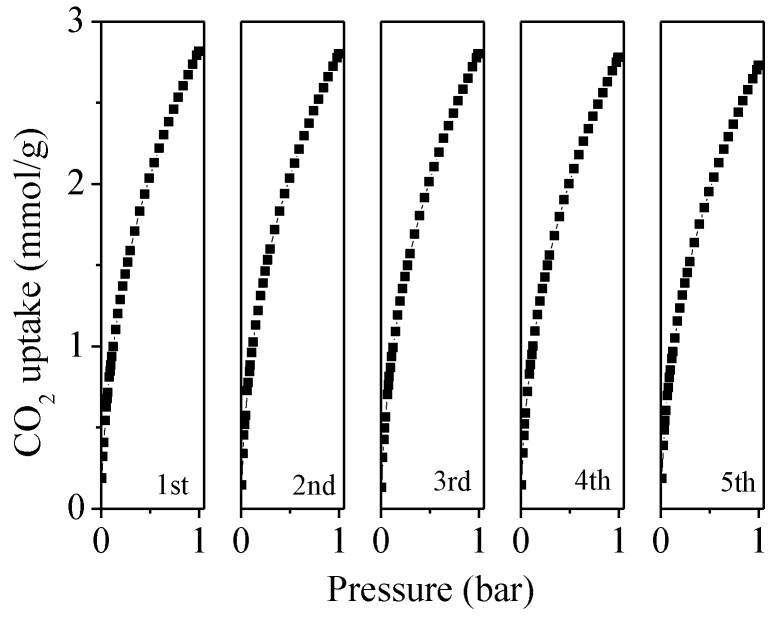
Cyclic study of CO_2_ adsorption for UFK-600-0.2 at 25 °C and 1 bar.

To further prove the practical usage of the as-prepared adsorbent, we examined the reversibility of CO_2_ adsorption on the optimal UFK-600-0.2 sample over five consecutive cycles at 25 °C and 1 bar. Before each test, the sorbent was heated at 200 °C for 6 h in a vacuum. As seen in Figure 8, 97% of initial CO_2_ uptake was maintained after 5 cycles, claiming facile recyclability, which could be recognized as a promising adsorbent for CO_2_ uptake performance.

## 3. Synthesis and Characterization

Commercial urea formaldehyde resin (UF) was used as the precursor, direct KOH activation of UF via a single step reaction was performed to obtained N-doped porous carbons. During activation process, three KOH/UF mass ratios i.e., 0.1, 0.2 and 0.3 and three activation temperatures i.e., 550, 600 and 650 °C were chosen. The as-achieved sorbents were assigned as UFK-T-m, of which T and m mean activation temperature and KOH/UF ratio, respectively. The yield of these N-doped porous carbons is in the range of 25–6%. The details about preparation, characterization of sorbents and CO_2_ adsorption performance measurement are recorded in the Supplementary Materials.

## 4. Conclusions

In conclusion, N-doped porous carbons were prepared by a facile one-pot KOH activating strategy from commercial urea formaldehyde resin (UF). The textural properties and nitrogen content of the N-doped carbos were carefully tuned by the activating temperature and KOH/UF mass ratios. As-prepared N-doped carbons show advanced porosity together with high N content. The optimal adsorbent presents a high CO_2_ adsorption capacity of 4.03 mmol/g at 0 °C and 1 bar. Moreover, as-prepared N-doped carbon adsorbents show multiple merits such as moderate isosteric heat of adsorption, high CO_2_/N_2_ selectivity, quick adsorption kinetics, good dynamic CO_2_ capture capacity and outstanding recycling performance. It has been pointed out that the CO_2_ uptake for these urea formaldehyde resin-derived N-enriched porous carbons was mainly determined by the textural feature of adsorbents, while the N content of carbons also plays a critical role to define the CO_2_ adsorption performance. The current study provides a facile and cost-effective way to obtain N-doped carbon for CO_2_ capture application.

## Figures and Tables

**Table 1:** Porous textural, elemental compositions, and CO_2_ uptakes of adsorbents derived from Urea formaldehyde resin under different conditions.

Sample	S_BET_^a^ (m^2^/g)	V_0_^b^ (cm^3^/g)	V_t_^c^ (cm^3^/g)	N (wt%)	C (wt%)	H (wt%)	CO_2_ Uptake (mmol/g)	
							25 °C	0 °C
UFK-550-0.1	8	0.03	-	23.51	57.43	2.72	1.67	1.83
UFK-550-0.2	474	0.25	0.17	22.42	58.09	3.41	2.31	2.50
UFK-550-0.3	688	0.31	0.26	20.34	56.03	3.25	2.64	3.16
UFK-600-0.1	388	0.18	0.14	21.32	58.23	3.01	2.31	2.81
UFK-600-0.2	865	0.44	0.33	19.69	59.62	3.90	2.82	4.03
UFK-600-0.3	895	0.44	0.32	18.32	55.13	3.56	2.78	3.95
UFK-650-0.1	662	0.33	0.25	19.34	57.82	3.29	2.70	2.95
UFK-650-0.2	980	0.52	0.41	18.20	59.63	3.71	2.67	3.71
UFK-650-0.3	950	0.49	0.37	16.85	58.67	3.52	2.71	3.52

^a^ Surface area was calculated using the BET method at *P/P_0_* = 0.005–0.05. ^b^ Total pore volume at *P/P_0_* = 0.99. ^c^ Evaluated by the t-plot method.

## Data Availability

The data presented in this study are available on request from the corresponding author.

## Supplementary Materials

The following supporting information can be downloaded at: https://www.mdpi.com/article/10.3390/molecules27206816/s1, Table S1: N-species contributions in total N obtained from fitting of the N 1s XPS spectra, Table S2: Comparison of the CO_2_ adsorption (25 °C and 1 bar) for different sorbents. References [24,60,61,62,68,69,70,71,72,73,74,75,76] are cited in the supplementary materials.
