# Supplementary material for: One-Pot Synthesis of N-Rich Porous Carbon for Efficient CO2 Adsorption Performance
Source: Molecules. 2022 Oct 12;27(20):6816. doi: 10.3390/molecules27206816 (PMC9610260; doi:10.3390/molecules27206816)
Supplement: Supplementary file 1 [file molecules-27-06816-s001.zip › molecules-1958790-supplementary.pdf]

One-Pot Synthesis of N-rich Porous Carbon for efficient CO_2 adsorption performance

(Supplementary materials)

Qiyun Yu [1], Jiali Bai [1], Muslum Demir [2], Bilge Nazli Altay [3,4], Xin Hu [1,*], Wenhao Jiang [1], Linlin Wang [5,*]

[1]Key Laboratory of the Ministry of Education for Advanced Catalysis Materials, Zhejiang Normal University, Jinhua, Zhejiang, 321004, PR China

[2]Department of Chemical Engineering, Osmaniye Korkut Ata University, Osmaniye, 80000, Turkey

[3]College of Engineering Technology, Print and Graphic Media Science, Rochester Institute of Technology, Rochester, NY, United States

[4]Institute of Pure and Applied Sciences, Marmara University, Türkiye

[5]Key Laboratory of Urban Rail Transit Intelligent Operation and Maintenance Technology and Equipment of Zhejiang Province, College of Engineering, Zhejiang Normal University, Jinhua, Zhejiang, 321004, PR China

[*]Corresponding author's e-mail: huxin@zjnu.cn (X. H.), wanglinlin@zjnu.cn (L. W.); phone: 86-151-0579-0257; fax: 86-579-8228-8269.

KOH activation

In a typical preparation, 2 g urea formaldehyde resin (UF) was combined with a solution that contained 0.4g KOH. After stirring vigorously for 8 h, the mixture was left overnight to dry at 120 °C in an oven. Afterwards, the sample was heated to 200 °C and then hold at this temperature for 1 hour, then the temperature was elevated

to 600 °C and maintain at this temperature for 2 h. During the activation process, the heating rate is 5 °C/min and nitrogen flow rate is 400 mL/min. Following activation, the sorbent was rinsed with distilled water until the pH value of the filtrate was roughly 7. The wet sample was then dried at 150 °C under vacuum for 24 h. The obtained sample was denoted as UFK-600-0.2.

Characterization

Powdered X-ray diffraction (XRD) patterns were carried out on a PHILIPS PW3040/60 powder diffractometer using CuKα radiation (λ =0.15406nm). Scanning electron microscopy (SEM Hitachi S-4800) was used to observe the morphology of the samples of carbon materials. Further details of the pore structure were determined by transmission electron microscopy (TEM, JEOL-2100F) operated at 200 kV. The CHN elements were analyzed using a VarioEL III Elemental Analyzer. Nitrogen adsorption and desorption isotherms were measured on a Beishide 3H-2000PS2 sorption analyzer at -196°C. Ultrahigh-purity N_2 (99.999%, Shanghai Pujiang Gas Co., Ltd) was used for measurement. Before measurement, the samples were degassed in a vacuum at 200°C for at least 12h. The specific surface area (S_{BET}) was calculated according to the multipoint Brunauer-Emmett-Teller (BET) method from the adsorption data in the relative pressure range between 0.001 and 0.05. The total micropore volume (V_t) was deduced from the N_2 adsorption data by the t-plot method, and the total pore volume (V_0) was estimated from the adsorbed amount of liquid nitrogen at a relative pressure of 0.99. The pore size distribution was calculated using the density functional theory (DFT) method. In addition, X-ray photoelectron (XPS)

measurements were performed using an AXIS Nova spectrometer (Kratos Inc., NY, USA) equipped with a monochromatic Al Kα X-ray source (1486.6 eV). XPS survey spectra were recorded with a pass energy of 160 eV, and high-resolution spectra with a pass energy of 40 eV.

The CO_2 adsorption isotherms were measured using the Beshide 3H-2000PS2 sorption analyzer at 0°C and 25°C, respectively. Pure CO_2 (99.99%, Shanghai Pujiang Gas Co., Ltd) was used for adsorption. Prior to each adsorption experiment, the sample was degassed for 12 h at 200°C to remove the guest molecules from the pores. The volume of narrow micropores (with sizes <1 nm), V_n, was calculated from CO_2 adsorption at 0°C using the Dubinin–Radushkevich (D-R) equation. The measurements were repeated for each sample, until the values fell within ± 2% of each other.

Measurement of dynamic CO_2 uptake of the sorbents

The dynamic CO_2 uptake of the sorbents was tested on a fixed-bed reactor schematically illustrated in Scheme S1 at 1 bar and 25 °C. First, the sample was heated at 100°C for 1 h under N_2 at a flow rate of 20 mL/min. The gas flow was shifted from nitrogen to a 10% mixture of CO_2 in N_2 at a flow rate of 10 mL/min, when the sample temperature was lowered to 25°C. The effluent gases were monitored online using an Agilent 7820A gas chromatograph with a thermal conductivity detector (TCD). From the breakthrough curves, the dynamic CO_2 capture capacity on an adsorbent was calculated.

Measurement of CO_2 adsorption kinetics

The adsorption kinetics of CO_2 was measured in a thermogravimetric analyzer (NETZSCH STA 449C). In the kinetic analysis, the sample (~5 mg) was degassed under a He stream at 200°C for 1 h. Next, the temperature was cooled to the experimental temperature of 25°C. Then the CO_2 gas was fed into the test chamber with a flow rate of 50 mL/min and the weight variation with time was recorded.

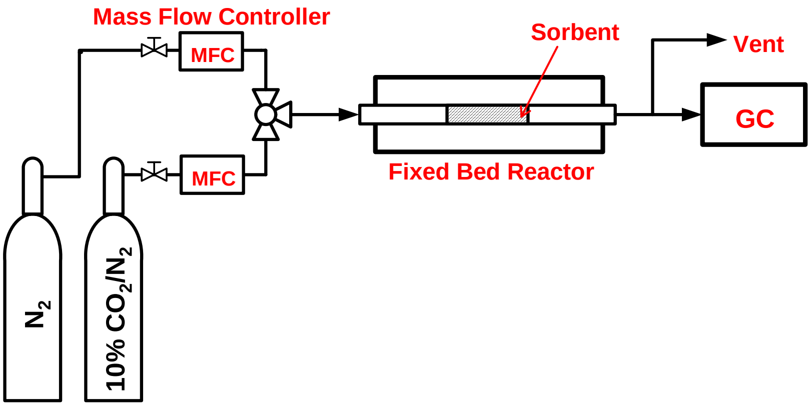

Scheme S1. Schematic of the fixed-bed reactor system.

Table S1. N-species contributions in total N obtained from fitting of the N 1s XPS spectra

Sample	N-6 (at. %)	N-5 (at. %)
UFK-600-0.2	43.03	56.97
UFK-600-0.3	42.12	57.88
UFK-650-0.3	42.47	57.53

Table S2. Comparison of the CO_2 adsorption (25 °C and 1 bar) for different sorbents

Sample	CO_2 uptake (mmol/g)	Ref.
G-900	1.4	S1
PCF-H5	2.3	S2
AA750	2.7	S3
GEPM-1	2.5	S4
GMNO-4	2.6	S5
GTCF-3	2.7	S6
MRF-2	2.5	S7
OM-CNS	3.0	S8
MOF-2	0.6	S9
MOF-505	3.3	S10
ZIF-78	2.7	S11
zeolite FAU	3.7	S12
Eggshell-CaO	4.5*	S13
UFK-600-0.2	2.8	This study

***** tested under 700°C

References

S1. Meng, L.-Y.; Park, S.-J., Effect of heat treatment on CO_2 adsorption of KOH-activated graphite nanofibers. *J. Colloid Interface Sci.* **2010,** 352, (2), 498-503.

S2. Li, L.; Wang, X.-F.; Zhong, J.-J.; Qian, X.; Song, S.-L.; Zhang, Y.-G.; Li, D.-H., Nitrogen-Enriched Porous Polyacrylonitrile-Based Carbon Fibers for CO_2 Capture. *Ind. Eng. Chem. Res.* **2018,** 57, (34), 11608-11616.

S3. Balahmar, N.; Mitchell, A. C.; Mokaya, R., Generalized Mechanochemical Synthesis of Biomass-Derived Sustainable Carbons for High Performance CO_2 Storage. *Adv. Energy Mater.* **2015,** *5*, (22), 1500867.

S4. Sui, Z.Y.; Cui, Y.; Zhu, J.H.; Han, B.H., Preparation of Three-Dimensional Graphene Oxide–Polyethylenimine Porous Materials as Dye and Gas Adsorbents. *ACS Appl. Mater. Interfaces* **2013,** *5*, (18), 9172-9179.

S5. Zhou, D.; Liu, Q.; Cheng, Q.; Zhao, Y.; Cui, Y.; Wang, T.; Han, B., Graphene-manganese oxide hybrid porous material and its application in carbon dioxide adsorption. *Chin. Sci. Bull.* **2012,** *57,* (23), 3059-3064.

S6. Zhou, D.; Cheng, Q.Y.; Cui, Y.; Wang, T.; Li, X.; Han, B. H., Graphene-terpyridine complex hybrid porous material for carbon dioxide adsorption. *Carbon* **2014,** *66*, 592-598.

S7. Zhou, H.; Xu, S.; Su, H.; Wang, M.; Qiao, W.; Ling, L.; Long, D., Facile preparation and ultra-microporous structure of melamine-resorcinol-formaldehyde polymeric microspheres. *Chem. Commun.* **2013,** *49*, (36), 3763-3765.

S8. Zheng, L.; Li, W. B.; Chen, J. L., Nitrogen doped hierarchical activated carbons derived from polyacrylonitrile fibers for CO2 adsorption and supercapacitor electrodes. *RSC Adv.* **2018,** *8*, (52), 29767-29774.

S9. Tian, Z.; Huang, J.; Zhang, X.; Shao, G.; He, Q.; Cao, S.; Yuan, S., Ultra-microporous N-doped carbon from polycondensed framework precursor for CO_2 adsorption. *Microporous Mesoporous Mater.* **2018,** *257*, 19-26.

S10. Millward, A. R.; Yaghi, O. M., Metal-Organic Frameworks with Exceptionally High Capacity for Storage of Carbon Dioxide at Room Temperature. *J. Am. Chem. Soc.* **2005,** *127*, (51), 17998-17999.

S11. Furukawa, H.; Yaghi, O. M., Storage of Hydrogen, Methane, and Carbon Dioxide in Highly Porous Covalent Organic Frameworks for Clean Energy Applications. *J. Am. Chem. Soc.* **2009,** *131,* (25), 8875-8883.

S12. Madhu, J.; Madurai Ramakrishnan, V.; Santhanam, A.; Natarajan, M.;

Palanisamy, B.; Velauthapillai, D.; Lan Chi, N. T.; Pugazhendhi, A., Comparison of three different structures of zeolites prepared by template-free hydrothermal method and its CO_2 adsorption properties. *Environ. res.* **2022,** 214, 113949.

S13. Hsieh, S.-L.; Li, F.-Y.; Lin, P.-Y.; Beck, D. E.; Kirankumar, R.; Wang, G.-J.; Hsieh, S., CaO recovered from eggshell waste as a potential adsorbent for greenhouse gas CO_2. *J. Environ. Management* **2021,** 297, 113430.
